# Projection of Future Extreme Precipitation and Flood Changes of the Jinsha River Basin in China Based on CMIP5 Climate Models

**DOI:** 10.3390/ijerph15112491

**Published:** 2018-11-08

**Authors:** Zhe Yuan, Jijun Xu, Yongqiang Wang

**Affiliations:** Changjiang River Scientific Research Institute, Changjiang Water Resources Commission of the Ministry of Water Resources of China; 430010 Wuhan, China; xujj07@163.com (J.X.); wangyq@mail.crsri.cn (Y.W.)

**Keywords:** climate change, extreme flood, CMIP5 climate models, Jinsha River Basin

## Abstract

Projecting future changes in extreme flood is critical for risk management. This paper presented an analysis of the implications of the Fifth Coupled Model Intercomparison Project Phase (CMIP5) climate models on the future flood in the Jinsha River Basin (JRB) in Southwest China, using the Xinanjiang (XAJ) hydrologic model. The bias-corrected and resampled results of the multimodel dataset came from the Inter-Sectoral Impact Model Intercomparison Project (ISI-MIP). Relatively optimal general circulation models (GCMs) were selected with probability density functions (PDFs)-based assessment. These GCMs were coupled with the XAJ model to evaluate the impact of climate change on future extreme flood changes in the JRB. Two scenarios were chosen, namely: a midrange mitigation scenario (Representative Concentration Pathway 4.5, RCP4.5) and a high scenario (RCP8.5). Results show that: (1) The XAJ model performed well in simulating daily discharge and was suitable for the study area, with *E_NS_* and *R*^2^ higher than 0.8; (2) IPSL-CM5A-LR and MIROC-ESM-CHEM showed considerable skill in representing the observed PDFs of extreme precipitation. The average skill scores across the total area of the JRB were 0.41 to 0.66 and 0.53 to 0.67, respectively. Therefore, these two GCMs can be chosen to analyze the changes in extreme precipitation and flood in the future; (3) The average extreme precipitation under 20- and 50-year return period across the JRB were projected to increase by 1.0–33.7% under RCP4.5 and RCP8.5 during 2020 to 2050. The Upper basin is projected to experience the largest increase in extreme precipitation indices, possibly caused by a warmer climate. The extreme flood under 20- and 50-year return period will change by 0.8 to 23.8% and −6.2 to 28.2%, respectively, over this same future period. Most of scenarios projected an increase during the near future periods, implying the JRB would be likely to undergo more flooding in the future.

## 1. Introduction

Flooding is a climate-related disaster. Between 1980 and 2013, economic losses caused by floods have exceeded $1 trillion (2013 values). Over this same period, more than 220,000 people have lost their lives [1]. Global climate change has already begun to affect the earth’s hydrologic cycle. Additionally, warmer climate will bring about high atmospheric moisture content, which will further lead to the increase of extreme precipitation events [2,3,4]. As flood is influenced greatly by precipitation, the risk of this kind of extreme hydrologic event is likely to grow. Thus, investigating flood changes in the context of climate change is imperative for risk management.

Most of the research on climate change impact use models for exploring ‘what-if’ questions. The main train of the research on flood risk management in regional climate change impact assessment is to do assessment of the impact of climate change on extreme precipitation and extreme streamflow with the aid of general circulation models (GCMs) output [5,6,7]. In order to make the coarse scale GCM data become suitable for hydrological simulation during this process, methods such as bias correction and statistical or dynamic downscaling have been brought in. Each step of the regional climate change impact assessment of flood may bring out uncertainty. Uncertainty will result from different GCMs, hydrological models, bias correction and downscaling methods. Previous studies have found that most of the uncertainty comes from the climate models and future emission scenarios. Unfortunately, this kind of uncertainty is innate and inevitable [8,9,10,11]. As a result, during the research of vulnerability and adaptation to climate change, the selection of climate models and emission scenarios should be treated integrally. A suite of GCMs has been provided by the Fifth Coupled Model Intercomparison Project (CMIP5). Scenarios describe how Greenhouse Gas (GHG) emissions could evolve until 2100, depending on various hypotheses. In 2014, the Intergovernmental Panel on Climate Change (IPCC) published the AR5, in which a new set of scenarios was defined: the Representative Concentration Pathways (RCPs). Although the uncertainly brought about by emission scenarios is innate and cannot be avoided, the same phenomena and consistent trend can be illustrated by most climate models. Moreover, the realization and structure of different GCMs will carry a certain amount of uncertainty [12,13]. Thus, it is essential to select a subset of GCMs for a specific application, which will contribute to the projection in future climate. 

Statistics like means and standard deviations in monthly, seasonal or yearly scales have been extensively applied to evaluate the ability of climate models. However, the biases or systematic errors identified in daily and peak data may be hidden in long averages (e.g., month, season and year). Moreover, means and standard deviations have limitations in representing the entire data distribution characteristic. A GCM might perform well in a mean value simulation, however, this does not mean it can capture other attributes [14,15]. Evaluating GCMs based on the probability density function (PDF) has one major advantage. If a GCM can simulate an entire PDF, this demonstrates its capability to simulate both extreme and common values, and thus, we might have more confidence in projections provided by this model [16]. Perkins et al. have carried out a simple method (skill score) to describe the relative similarity between model and observed PDFs. It is clearer, more robust and reliable than classical statistical tests. Different variables can be directly compared using this method [17]. This research mainly focuses on the impact of climate change on flood; thus, the simulation capacity of GCMs on extreme precipitation is the key factor in determining the reliability of the whole research. Hence, PDF-based assessment was used to assess different climate models.

Water resources in the Jinsha River Basin (JRB) are distributed unevenly in both the temporal and spatial scale, which results in frequent floods occurring in wet season. Additionally, the extreme precipitation is expected to increase in the future [18,19], which may lead to higher risk of flood. It is of great importance to investigate the effects of climate change on extreme floods in this region. Thus, this research takes the JRB as the study area. A PDF-based assessment method was utilized to select the relatively optimal GCMs. With the aid of climate models and a hydrologic model, future changes of extreme precipitation and floods in the context of climate change were projected. This research will provide the policymakers with scientific proof on changes in flood, so as to make decisions on the change of magnitudes of design floods.

## 2. Materials and Methods

### 2.1. Study Area

**Figure 1:**
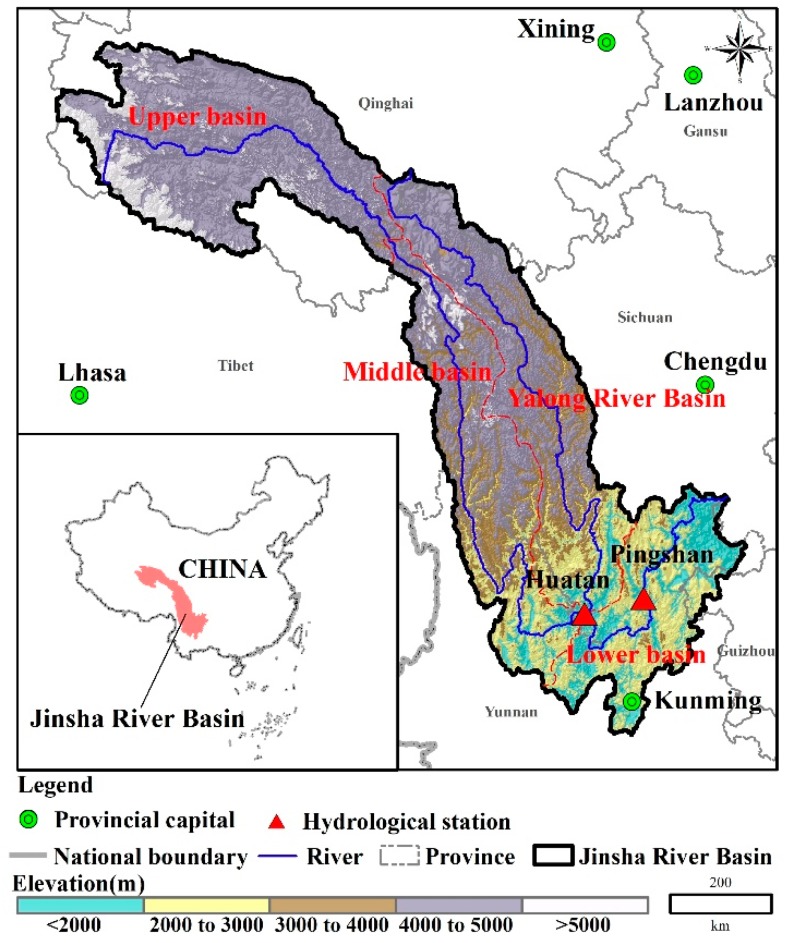
The location of the Jinsha River Basin (JRB).

The Jinsha River Basin (JRB, 90°30′–105°15′ E and 24°36′–35°44′ N) has a total area of 473,200 km^2^, which is about 26% of the total drainage area of the Yangtze River Basin [20]. The Jinsha River runs through four provinces and an autonomous region (namely, Qinghai, Tibet, Yunnan, Sichuan and Guizhou). Originating from the peak of east Geladan Snowy Mountain in the Tanggula Mountains, the Jinsha River flows through the Western Sichuan Plateau, Hengduan Mountains and Yunnan-Guizhou Plateau to the mountain area of Southwest Sichuan, with a total length of 3464 km [21]. The Jinsha River’s longest tributary is the Yalong River, whose total length is 1187 km (Figure 1). According to the data set provided by Data Center for Resources and Environmental Sciences, Chinese Academy of Sciences (RESDC), the total population in the JRB area was about 24 million in 2010, of which about 6.6 million in the Sichuan Province, about 13.8 million in the Yunnan Province and about 0.8 million in the Guizhou Province. The population in Qinghai and Tibet is low. The average annual precipitation of the JRB is approximately 710 mm: the annual precipitation of the lower reaches is approximately 900–1300 mm. The middle and upper reaches are mountainous canyon regions with an average annual precipitation of 600–800 mm. The annual precipitation in the source area upstream is less than 200 mm. Precipitation is mainly concentrated from June to October, which accounts for about 75–85% of annual precipitation (wet season). The spatial distribution of the temperature is similar to the precipitation. The overall trend is increasing from upstream towards downstream and from northwest to southeast. The average annual temperature of the JRB area is below 0 °C (upper reach), approximately 5 °C (middle reach) and above 10 °C (lower reach), respectively. According to statistics of discharge time series of 1950–2011 at the Pingshan station, which can be considered as the controlling hydrologic station, the annual average runoff of the JRB is 143 billion m^3^. The Jinsha River flooding along the mainstream is mainly caused by extreme precipitation over a long time period. Heavy rainfall mainly happens at the middle and lower reaches of the Jinsha River. Floods generally occur in late June to mid-October, and more frequently, from July to September.

### 2.2. Data Sets

#### 2.2.1. Observed Hydro-Meteorological Data

**Figure 2:**
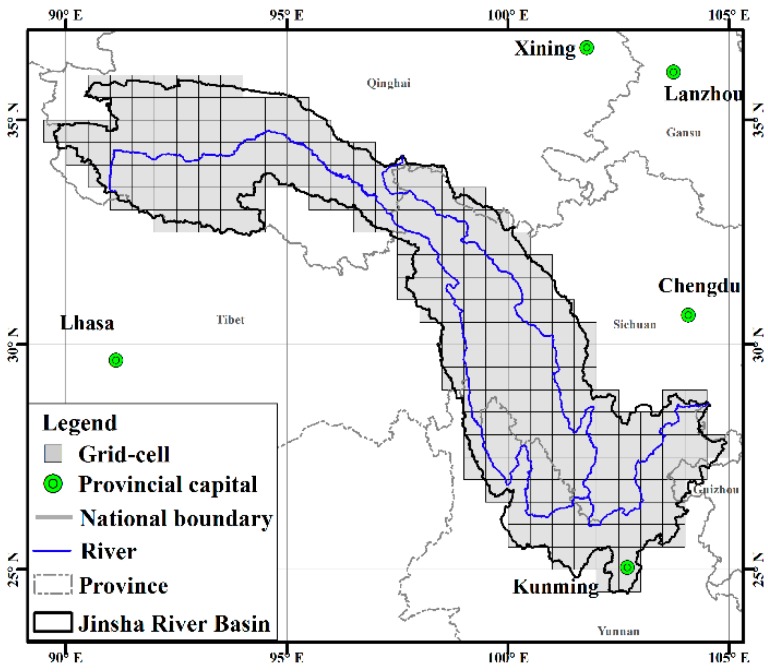
Grid cells in the JRB.

Observed daily precipitation, minimum and maximum temperature data was collected from China’s Ground Precipitation 0.5° × 0.5° Gridded Dataset (V2.0) and China’s Ground temperature 0.5° × 0.5° Gridded Dataset (V2.0) (http://data.cma.cn/), which was provided by the National Meteorological Information Center (NMIC) of the China Meteorological Administration (CMA). This dataset was based on the daily observations from 1961 to the present at 2474 national meteorological stations over the Chinese mainland. Data assessment showed that the gridded value had high correlation and little error with observation; thus, the gridded value can be used to reflect the variations of precipitation and temperature. In this study, 208 grid cells covering the JRB were selected (Figure 2).

Daily discharge data was collected from two hydrologic stations in the lower reach (Figure 1). It was used to calibrate/validate the hydrology model and calculate the extreme flow (see Section 2.3). The basic information of the hydrologic stations is listed in Table 1.

#### 2.2.2. Future Climate Change Scenarios

In this study, five global climate models in the IPCC AR5 report were selected: GFDL-ESM2M, HADGEM2-ES, IPSL-CM5A-LR, MIROC-ESM-CHEM and NORESM1-M. Information of each GCM is listed in Table 2. Each of these models offers continuous daily-series data, including historical simulations and future projection simulations forced under RCP2.6, RCP4.5, RCP6.0 and RCP8.5. These four RCP scenarios were named according to a possible range of global radiative forcing values in 2100 relative to pre-industrial values. It is assumed that the global radiative forcing will reach the highest point at ~3 W m^−2^ before 2100. If this value declines to 2.6 W m^−2^ by 2100, this corresponding scenario is RCP2.6; stabilizations without overshoot pathways to 6.0 and 4.5 W m^−2^ at stabilization after 2100 means RCP6.0 and RCP4.5; if this value rockets up to 8.5 W m^−2^ by 2100, the corresponding scenario is RCP8.5 [22]. The data was bias-corrected and resampled by the Inter-Sectoral Impact Model Intercomparison Project (ISI-MIP, http://www.isi-mip.org). It covers the period from 1960 to 2099 on a horizontal grid with 0.5° × 0.5° resolution [23]. The data was bias-corrected to make sure the consistency of the long-term statistical characteristic of the whole data and the observed data from 1960–1999 [24]. This bias-correction method was specifically developed for this project to keep the absolute trends in temperature and relative trends in precipitation and the other variables [25]. There are 208 boxes in and around the JRB and the spatial resolution of data can meet the requirements of hydrological simulation in the JRB. Since this research is focusing on extreme precipitation and discharge, RCP4.5 (medium) and RCP8.5 (high) scenarios were chosen to project the extreme precipitation and discharge variation.

### 2.3. Methodology

#### 2.3.1. XAJ Hydrologic Model

The Xinanjiang (XAJ) model was used in this study for daily discharge simulation. The XAJ model has been widely used in simulating rainfall-runoff, forecasting flood and planning water resources in humid and semi-humid regions [31]. It provides an integral structure to statistically describe the non-uniform distribution of runoff producing areas. It has been proved that, due to its description of vertical spatial distribution of soil moisture storage, the XAJ model performs better than other models [32,33]. Further detailed information relating to the XAJ model can be obtained from relevant references [34,35]. The model is driven by daily precipitation (*P*) and potential evapotranspiration (*PE*). In this study, the daily PE in the XAJ model was calculated by using the Hargreaves method based on daily maximum and minimum temperature [36]. The areal daily precipitation and potential evapotranspiration act as input to drive the XAJ model, and can be evaluated through Equation (1). The parameters of the XAJ model were calibrated by fitting the calculated daily discharge against the observed data. Correlation coefficient of linear regression equation (*R*^2^) and Nash-Sutcliffe values (*E_NS_*) [37] were chosen to quantify the model’s performance of simulating daily discharge.

(1) $$P=\frac{\sum_{i}^{n}P_{i}S_{i}}{\sum_{i}^{n}S_{i}},PE=\frac{\sum_{i}^{n}PE_{i}S_{i}}{\sum_{i}^{n}S_{i}}$$

where *P* and *PE* are the areal precipitation and potential evapotranspiration, respectively; *P_i_* and *PE_i_* are the precipitation and potential evapotranspiration of the grid box *i*, *Si* is the area of grid box *i*; *n* is the number of grid boxes in the Huatan catchment or Pingshan catchment.

#### 2.3.2. Extreme Precipitation and Flood Indices

The multi-day extreme precipitation and discharge indices, widely used in extreme events analyses [38], were selected to reflect extreme precipitation and flow in this study (Table 3). One type of index measures the annual maximum amount of daily precipitation, such as AMX1p, AMX3p and AMX7p. Another type of index measures the maximum discharge in successive *n* days, such as AMX1d, AMX3d and AMX7d.

A physically more meaningful and more relevant quantity for risk assessment is the probability of the variable (here, multi-day extreme precipitation and discharge) exceeding a certain level. The precipitation and discharge of annual maximum series under given return periods were calculated based on the generalized extreme value (GEV) distribution, which is widely utilized in modeling extreme events in meteorology and hydrology [19]. Equations (2) and (3) are the distribution function:

(2) $$\begin{array}{l} F\left(x\right)=\exp\left\{-{\left[1+k\left(\frac{x-\xi }{\alpha }\right)\right]}^{-\frac{1}{k}}\right\},k\neq 0\\ F\left(x\right)=\exp\left\{-\exp\left[-\left(\frac{x-\xi }{\alpha }\right)\right]\right\},k=0 \end{array}$$

where, *ξ*, *α* and *k* are parameters standing for location, scale and shape, respectively. In this study, the parameters were estimated by using the Maximum Likelihood Estimation method [39].

The flood magnitude (*x_T_*) for *T*-year return period can be calculated as follows:

(3) $$x_{T}=\xi +\frac{\alpha }{k}\left\{1-{\left[-\ln\left(1-\frac{1}{T}\right)\right]}^{k}\right\}$$

In this study, return periods of 20 and 50 years were calculated and analyzed.

#### 2.3.3. Selection of GCMs and Projection of Future Extreme Flood Changes

**Figure 3:**
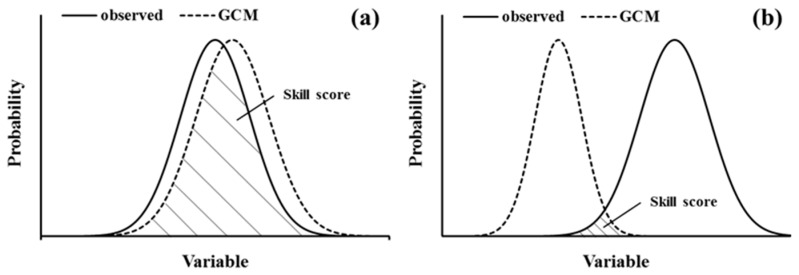
Diagrams of the general circulation model (GCM) vs the observed probability density function (PDF), illustrating the total skill score in (**a**) a near-perfect skill score and (**b**) a very poor skill score.

Coupled climate models were evaluated and relatively optimal GCMs were selected to do hydro-meteorological variability projection. The simulation of AMX1p, AMX3p and AMX7p in 208 cells of the JRB were evaluated based on probability density functions (PDFs). An alternative metric-skill score (*SS*) was defined to illustrate the similarity between two PDFs. The cumulative minimum value of two distributions of each binned value can be calculated with the *SS*, which means the overlapping area between two PDFs. The skill score (*SS*) is greater than 0 and smaller than 1. When *SS* is close to 1, it means that the simulated value fits perfectly with the observed value (Figure 3a). When *SS* is close to 0, it means the overlap between simulated value and observed value is negligible (Figure 3b) [17]. This can be seen in Equation (4):

(4) $$SS=\sum_{1}^{n}\min\left(Fs_{n},Fo_{n}\right)$$

where, *n* stands for the number of bins; *Fs_n_* stands for the frequency of values in a given bin from the model; and *Fo_n_* stands for the frequency of values in a given bin from the observed data. Adding up the minimum frequency values over all bins and *SS* can be obtained. In this research, GEV was utilized to obtain PDFs of the observed and simulated value.

The bias was chosen in this research to assess the reconstructing capacity of GCMs to rebuild the precipitation characteristics, so as to test the rationality of using skill score to assess climate models. Specific equations are listed as follows:

(5) $$B=\left|\frac{\bar{P_{s}}-\bar{P_{o}}}{\bar{P_{o}}}\right|$$

where, *B* is the absolute value of relative deviation; $\bar{P_{s}}$ and $\bar{P_{o}}$ are the simulated and observed multi-year average extreme precipitation; *B* is greater than 0 and smaller than 1; the closer *B* is to 0, the better the performance of the GCM.

Driven by the baseline, daily climatology and the projected future daily climate data sets coming from the relatively optimal GCMs, the calibrated XAJ model can output the simulated daily discharge in both baseline (1961 to 1990) and future period (2020 to 2050). With the parameterized GEV distribution in different periods, relative changes in AMX*n*p and AMX*n*d under the 20- and 50-year return period can be estimated and represent changes in discharge in the future.

## 3. Results and Analysis

### 3.1. Historical Discharge Simulations with XAJ Model

**Figure 4:**
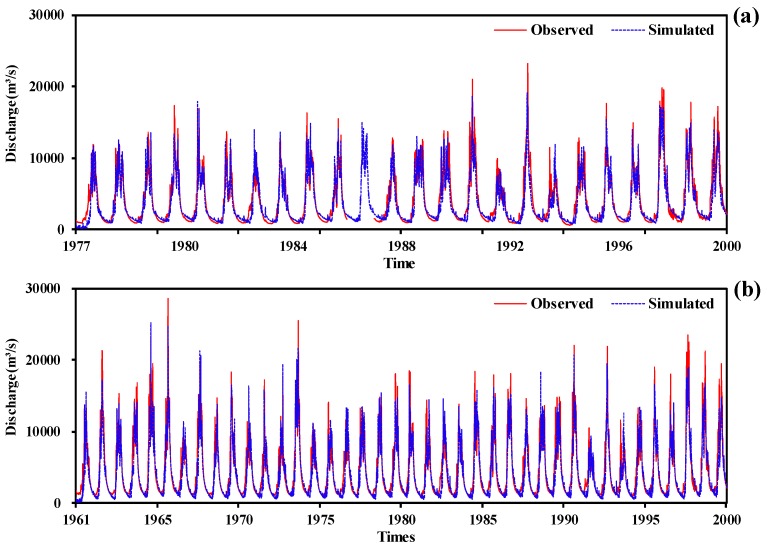
Observed and simulated daily discharge: (**a**) the Huatan station; (**b**) the Pinghshan station.

Based on the 208 cells with daily precipitation, maximum and minimum temperature, input driving data of the XAJ model were generated. The recorded data series was divided into calibration period (before 1990) and validation period (after 1990). Figure 4 shows the simulated and observed daily discharge in the calibration period and validation period. Table 4 summarizes the assessment of the XAJ model in the Huatan and Pinghshan stations. In Table 4, it can be seen that *E_NS_* and *R*^2^ are both higher than 0.8 in both calibration and validation period, which means fair performance of the XAJ model. Generally, a daily *E_NS_* of 0.65 or higher means the simulation is very good [40]. That is to say, the established XAJ model was acceptable to do daily discharge simulation in the JRB.

### 3.2. Comparison of GCM Simulations with Observations

**Figure 5:**
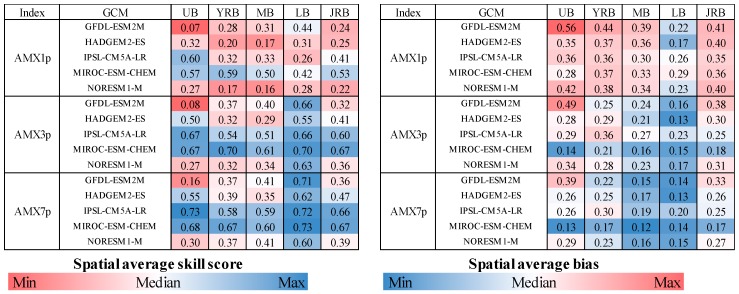
The Spatial average skill score (**left**) and bias (**right**) for the five GCMs. Note: UB-Upper basin; YRB-Yalong River Basin; MB-Middle basin; LB-Lower basin; JRB-Jinsha River Basin.

With the observed PDF and GCM PDF for each grid square during 1961 to 2000, the *SS*s estimated for individual cells can be calculated. Average values of *SS* and *B* over four sub-catchments (namely, the Upper basin, Yalong River Basin, Middle basin, Lower basin and the Jinsha River Basin) are illustrated in Figure 5. Although several variations exist among different models, the five GCMs in this study performed better in the Lower basin than that in the Middle and Upper basins and better for AMX3p and AMX7p than AMX1p. Compared to the other three GCMs, IPSL-CM5A-LR and MIROC-ESM-CHEM were capable of capturing the characteristics of extreme precipitation statistical distribution and average value, especially for AMX3p and AMX7p. Taking the AMX3p as an example, the average *SS* (*B*) across the JRB for IPSL-CM5A-LR and MIROC-ESM-CHEM were 0.60 (0.25) and 0.67 (0.18), respectively, which were superior to the projection of other models. Based on the above analysis, the outputs that came from IPSL-CM5A-LR and MIROC-ESM-CHEM were chosen for the analysis of future extreme flood changes.

### 3.3. Changes in Extreme Precipitation for 2020 to 2050

**Figure 6:**
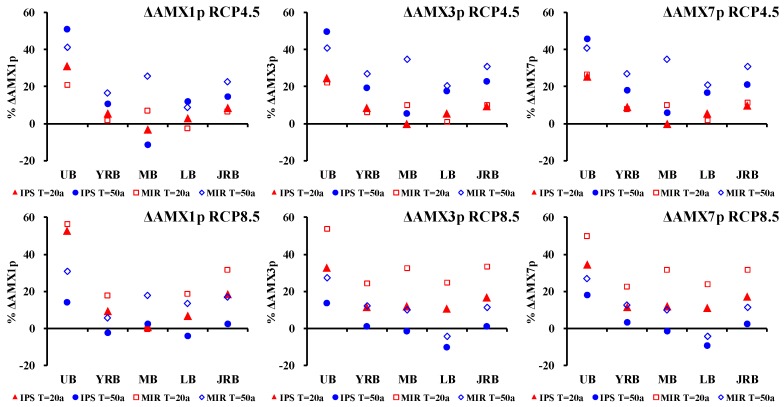
The projected changes to average AMX*n*p-*T*yr (*n* = 1, 3, 7; *T* = 20a, 50a) of Upper basin (UB), Yalong River Basin (YRB), Middle basin (MB), Lower basin (LB) and the whole Jinsha River Basin (JRB) with respect to the reference period (1961 to 1990) derived from the projections of RCP4.5 and RCP 8.5 climate scenarios of IPSL-CM5A-LR and MIROC-ESM-CHEM.

Flood is most likely to be triggered by extreme precipitation [41,42]. Hence, extreme precipitation events variations (AMX1p, AMX3p and AMX7p) from the reference period (1961 to 1990) and the projection period (2020 to 2050) were firstly analyzed. The calculation of relative change between simulated value in projection period and that in the reference period could illustrate the evolution trend, and thus, eliminate the impacts of systematic deviation caused by GCMs. Only the above selected optimal GCMs (IPSL-CM5A-LR and MIROC-ESM-CHEM) were utilized for the analysis of the future extreme precipitation, so as to reduce the uncertainty from the GCMs. Figure 6 shows the relative changes of the average AMX*n*p under 20-year return period (*P* = 5%) and 50-year return period (*P* = 2%) in the four sub-basins and the whole JRB between the baseline and future scenarios. The two relatively optimal GCMs project a consistent increase in AMX*n*p-*T*yr (*n* = 1, 3, 7; *T* = 20a, 50a) by 1.0–33.7% across the JRB under RCP4.5 and RCP8.5 from 2020 to 2050. Among the four sub-basins of JRB, the upper basin will face the largest increase in AMX1p (14.4 to 56.3%) and AMX3p (13.9–53.9%) and AMX7p (18.0 to 50.1%). These results indicate that in the near 30 years, the JRB, especially the upstream basin, will suffer from intensified extreme floods.

**Figure 7:**
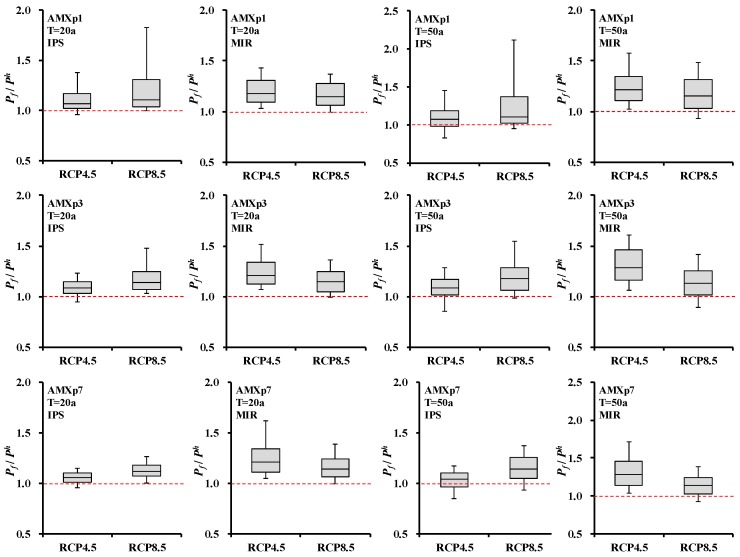
Boxplot of AMX*n*p-*T*yr (*n* = 1, 3, 7; *T* = 20a, 50a) change in each grid-box for different RCPs and GCMs. *Y*-axis is the ratio of projected value (*P_f_*) to historical value (P_h_). *P_f_*/*P_h_* > 1 means the increase of AMX*n*p; *P_f_*/*P_h_* < 1 means the decrease of AMX*n*p. The box portion represents the 25th, 50th to 75th percentile, and the whiskers represent the 10th percentile and the 90th percentile.

**Figure A1:**
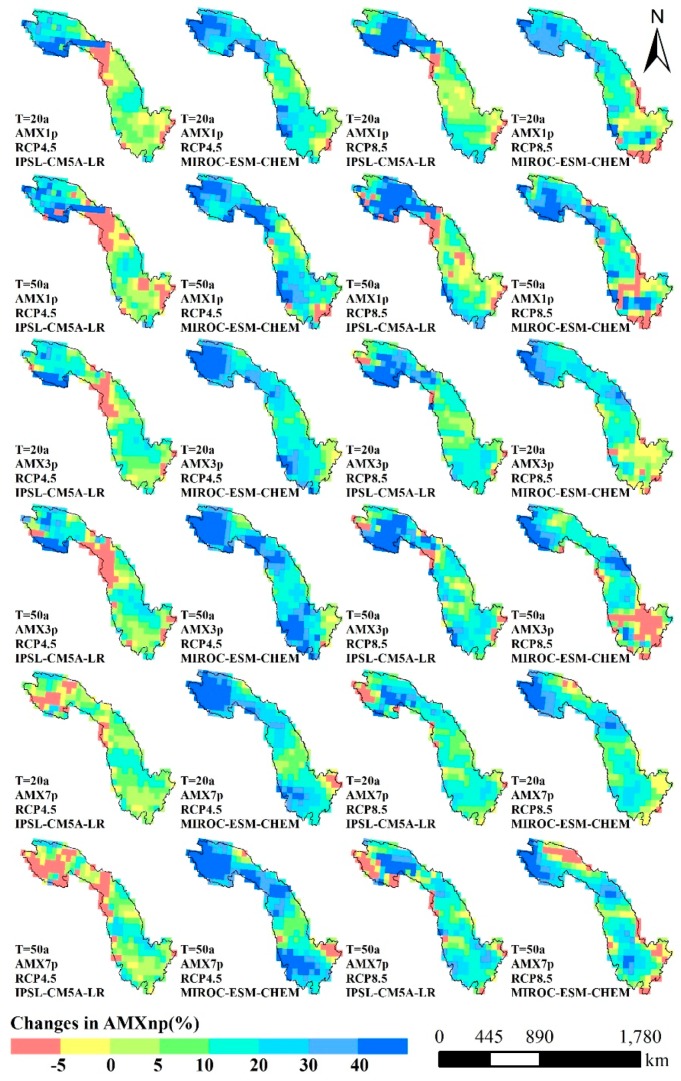
Changes in AMX1p, AMX3p and AMX7p with 20-/50-year return period from baseline to future.

According to the spatial distribution of extreme precipitation shown by Figure A1, Boxplots of AMX*n*p change in each grid-box can be obtained (Figure 7). Thus, the spatial difference of extreme precipitation in different combined condition of GCMs and RCPs can be quantitatively described (longer box means bigger difference). It can be seen that in RCP4.5, spatial difference of extreme precipitation projected by IPSL-CM5A-LR was relatively small: extreme precipitation variation in grid-box ranges from 0 to 15%. While the spatial difference of extreme precipitation projected by MIROC-ESM-CHEM was relatively big: extreme precipitation variation in grid-box ranges from 10% to 35%; in the meantime, the precipitation variation was bigger than that projected by IPSL-CM5A-LR. In RCP8.5, extreme precipitation variation projected by both IPSL-CM5A-LR and MIROC-ESM-CHEM will range from 5% to 25%.

### 3.4. Changes in Extreme Floods for 2020 to 2050

**Figure 8:**
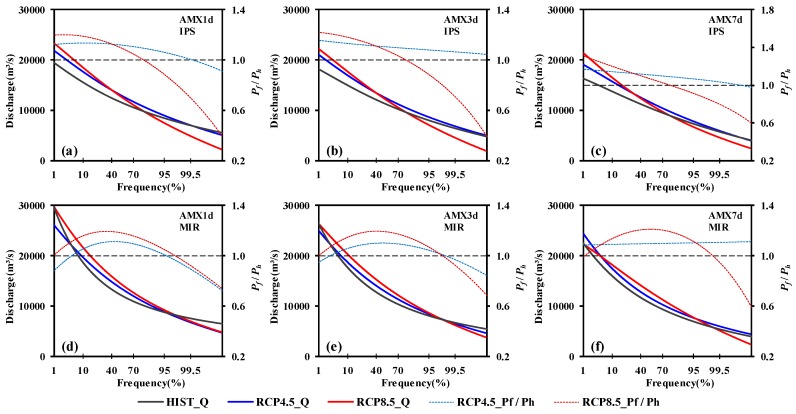
Generalized extreme value (GEV) frequency distributions of AMX*n*d in Pingshan under different climate change scenarios. HIST_Q, RCP4.5_Q and RCP8.5_Q means the value of AMX*n*d. RCP4.5_*P_f_*/*P_h_* and RCP8.5_ *P_f_*/*P_h_* means the ratio between projected value (*P_f_*) and historical value (*P_h_*). *P_f_*/*P_h_* > 1 means increasing AMXnd while *P_f_*/*P_h_* < 1 means decreasing AMX*n*d.

Figure 8 illustrated the GEV frequency distributions of AMX*n*d in Pingshan, which can be used to compare the frequency of extreme floods between baseline and future periods. An increase trend has been detected in most climate change scenarios when frequency was less than 10%. However, in the same frequency range, variation of AMX*n*d projected by IPSL-CM5A-LR and MIROC-ESM-CHEM showed a significant difference. In RCP4.5, AMX*n*d projected by IPSL-CM5A-LR varied between 12 to 17%, while AMX1d, AMX3d and AMX7d projected by MIROC-ESM-CHEM were −12 to 6%, −5 to 6% and 8 to 9% respectively. When the frequency was less than 2%, AMX1d and AMX3d showed a decreasing trend; in RCP8.5, AMX*n*d projected by both IPSL-CM5A-LR and MIROC-ESM-CHEM showed an increasing trend; 18 to 30% and 0 to 15%, respectively.

**Figure 9:**
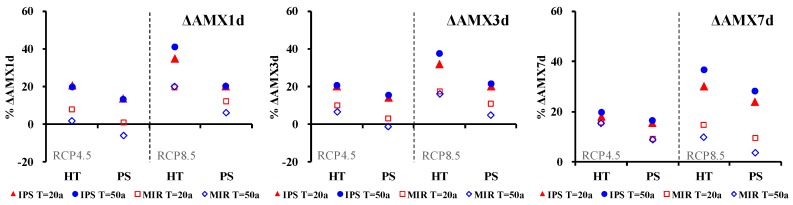
The projected changes of average AMX*n*d-*T*yr (*n* = 1, 3, 7; *T* = 20a, 50a) of Huatan (HT) and the Pingshan (PS) with respect to the reference period (1961 to 1990) derived from the projections of RCP4.5 and RCP 8.5 climate scenarios of IPSL-CM5A-LR and MIROC-ESM-CHEM.

To further investigate the relative changes in AMX*n*d, two different return periods (20-year and 50-year) were chosen. As shown in Figure 9, the largest percentage increases in AMX*n*d were mainly found for the RCP8.5 scenario of IPSL-CM5A-LR in 2020–2050. Take the 50-year return period as an example: the AMX1d, AMX3d and AMX7d would increase by 12.9%, 15.0% and 16.3%, respectively, in the Pingshan Station. The decrease of AMX1d and AMX3d were found for the RCP4.5 scenario of MIROC-ESM-CHEM in the Pingshan Station (−6.2% in AMX1d and −1.6% in AMX3d for the 50- year return period). AMX*n*d-*T*yr (*n* = 1, 3, 7; *T* = 20a, 50a) showed an increasing trend in all the other combination situation of scenarios and GCMs. The range of relative changes in Pingshan are described here, when the results from the RCP4.5 and RCP8.5 scenarios of the two relatively optimal GCMs are considered.. When considering the results in RCP4.5 and RCP8.5 scenarios of the two relatively optimal GCMs, the range of relative changes in Pingshan were described as follows. For AMX1d, the relative changes ranged from 0.8 to 19.9% for the 20-year return period and from −6.2 to 20.1% for the 50-year return period. For AMX3d, the relative changes ranged from 2.9 to 19.9% for the 20-year return period and from −1.6 to 21.4% for the 50-year return period. For AMX7d, the relative changes ranged from 9.0 to 23.8% for the 20-year return period and from 3.6 to 28.2% for the 50-year return period.

## 4. Discussions

The future extreme precipitation and flood variation of the JRB were analyzed in this study. Predominantly, an increase of projected extreme precipitation and flood has been informed by the GCMs. Most of these findings are consistent with previous studies. Both Huang et al. and Zhang et al. found an increasing trend in extreme precipitation indices in the upper Yangtze reaches [18,43]. Su et al. found an increase of 0.3 to 13.1% for peak discharge in the upper Yangtze River Basin during 2036 to 2065 [44]. The study of Gu et al. also showed that flood frequency and magnitude in the upper Yangtze River Basin are likely to increase significantly in the future. The AMX1d and AMX7d with return periods of 50, 20 and 10 years may turn into hydrological extreme events with return periods of approximately 15, 7 and 3 years [45]. There has been evidence showing that in the next decades, the JRB is likely to suffer from more floods.

In this study, we used multiple GCMs and RCP scenarios and one hydrological model to discuss the possible variation in extreme floods. Although we have assessed the performance of multiple climate models and selected the most suitable climate models for this research before analyzing the future variation of flood, uncertainty caused by future climate conditions still exist in future flood projection. Similar research was carried out by Bell et al. and Kay et al. [46,47]. Another uncertainty source is the structure and parameters of the hydrological models. Since the impact of this kind of uncertainty is much less than that in GCMs [8,48], this research did not take the uncertainties from hydrological models into consideration. Still, this does not mean that this kind of uncertainty should be totally ignored. Taking the XAJ model used in this study as an example, the potential evapotranspiration (PET) can be estimated by the Penman-Monteith method or Hargreaves method. More meteorological data, such as solar radiation, is needed when using Penman-Monteith method, while Hargreaves method only needs temperature data. Since the data used by the XAJ model in this research is the gridded dataset provided by National Meteorological Information Center, which only involves precipitation and temperature, Hargreaves method was utilized to calculate PET. As a result, the projection of future flood did not take variation of solar radiation, wind speed or relative humidity into consideration. Furthermore, we assumed that the hydrological model parameters calibrated from observation are still valid when they are used in future climate simulations. It is on this basis that the impact of climate change could be evaluated by a hydrological model [49]. Actually, as pointed by Merz et al., hydrological model parameters may potentially change if calibrated to different periods [50]. Therefore, this study will do further research on the uncertainty produced by model structure and its parameters. Different hydrological models and PET computation methods shall be used to compare differences of response to climate change in the JRB.

The data of climate change scenarios used in this study is provided by Inter-Sectoral Impact Model Intercomparison Project, which has been bias-corrected and resampled with 0.5° × 0.5° resolution, which also means that the original GCMs data has been statistically downscaled. Compared with the dynamical downscaling method based on Regional Climate Model (RCM), statistical downscaling method lacks solid mathematical and physics foundation. Thus, projected flood variation using the data in this research may have difference in the flood projection using RCMs’ output data. In the further research, RCMs such as PRECIS could be used to analyze the impact of climate change on extreme precipitation and flood in JRB and the result of two studies could be compared. Due to the daily precipitation provided by GCM, research on hourly flood peak is hard to carried out using this data set to analyze the impact of climate change on flood. Thus, annual maximum *n*-day discharge on hydrologic station was chosen to characterize the impact of climate change on flood. In the meantime, the hydrological model (the XAJ model), which can describe the relationship between rainfall and runoff well, was selected. However, the impact of climate change on designed flood hydrograph was the most concerned issue in flood risk management. In the following research, time set method can be utilized to obtain precipitation data with shorter temporal scale (such as hourly scale) based on the GCMs’ output. Based on this, the storm event watershed model, e.g. the FLOW-R2D model, can be used to do hydrodynamic simulation, so as to assess the impact of climate change on future typical flood propagation [51,52,53].

## 5. Conclusions

This research studied the projected extreme precipitation and flood variation of the JRB in RCP4.5 (medium) and RCP8.5 (high) scenarios based on five CMIP5 GCMs. The XAJ model was employed to simulate daily discharge, using 0.5° × 0.5° grid cells across the JRB for the baseline (1961 to 1990) and for the future period (2020 to 2050). Six extreme indices (AMX*n*p and AMX*n*d, *n* = 1, 3, 7) were chosen for the analysis.

Since *E_NS_* and *R*^2^ were both higher than 0.8, it was suggested that the XAJ model performed well in simulating the daily discharge. This model can be used to estimate the impact of climate change on future flood in the JRB. Both the PDF-based assessment and model bias were used to evaluate the GCMs performance in the JRB. In general, IPSL-CM5A-LR and MIROC-ESM-CHEM showed considerable skill in representing the observed AMX*n*p, which can be chosen as the relatively optimal GCMs to analyze the changes in extreme precipitation and flood in the future.

As expected, extreme precipitation indices derived from precipitation projections of the GCMs for JRB exhibit a wide range of possible changes. The average AMX*n*p (*P* = 2% and *P* = 5%) across the JRB were projected to increase by 1.0–33.7% during 2020 to 2050. However, in different combination of climate models and scenarios, extreme precipitation showed a unanimous increasing trend, indicating that extreme precipitation will occur more frequently and more severe in the JRB in the future. According to the extreme precipitation projection revealed in this research, the Upper basin may suffer from the largest increase in extreme precipitation. This phenomenon may be caused by the melting and evaporation of glacier in the mountain area of Yangtze River source region due to global warming.

The projection of extreme floods indicates that AMX1d, AMX3d and AMX7d showed very similar trends during the near future periods (2020–2050). The largest percentage increases in AMX*n*d were found for the RCP8.5 scenario of IPSL-CM5A-LR in 2020–2050, whereas the AMXnd will increase slightly and may even decrease for the RCP4.5 scenario of MIROC-ESM-CHEM. There is a relatively large variability in the projected ranges of AMX*n*d under the different future climate change scenarios. However, most of scenarios projected an increase during the near future periods (compared to the baseline 1961 to 1990). Overall, the percentage changes in AMX1d, AMX3d and AMX7d under 50-year return period ranged from −6.2 to 20.1%, −1.6 to 21.4% and 3.6 to 28.2%, respectively.

This research is a preliminary assessment of future extreme precipitation and flood in the JRB. Although uncertainties unavoidably exist in the projections, the results should be taken with care because the extreme floods of the JRB would be likely to become more frequent and intense in the next several decades. Furthermore, combining this research with the existing hydraulic engineering conditions, potential submerged area caused by extreme flood could be simulated. Then, the affected inhabitants, buildings and agricultural land could be identified, so as to provide scientific support for the drawing of flood risk map in the context of climate change.

## Figures and Tables

**Table 1:** Basic information of the two hydrologic stations.

Hydrologic Station	Lon. (E°)	Lat. (N°)	Catchment Area (10^3^ km^2^)	Area Percent (%)	Data Period
Huatan	102.88	26.88	429.2	90.7	1977–2000
Pingshan	104.17	28.63	459.0	97.0	1950–2011

**Table 2:** The five models of the Fifth Coupled Model Intercomparison Project Phase (CMIP5) characteristics.

Centre	Country	Name	Description
Geophysical Fluid Dynamics Laboratory (GFDL)	United States	GFDL-ESM2M	Based on CM2.1 ^a^, coupled by AM2 ^b^ (Atmosphere), LM3.0 ^c^ (Land), SIS ^d^ (Sea ice) and MOM4p1 ^e^ (Ocean) [26].
Hadley Centre for Climate Prediction and Research, Met Office	United Kingdom	HADGEM2-ES	Building on HadGEM2-AO ^f^ by including the TRIFFID ^g^ dynamic vegetation model (terrestrial ecosystem), the Diat-HadOOC ^h^ model (Oceanic ecosystem) and the UKCA model ^i^ (Tropospheric chemistry) [27].
L’Institut Pierre-Simon Laplace (IPSL)	France	IPSL-CM5A-LR	Coupled by LMDZ5A ^j^ (Atmosphere), ORCHIDEE ^k^ (Land), LIM-2 ^l^ (Sea ice) and NEMOv3.2 ^m^ (Ocean) [28].
Technology, Atmosphere and Ocean Research Institute, and National Institute for Environmental Studies	Japan	MIROC-ESM-CHEM	On the basis of MIROC, including an atmospheric chemistry component (CHASER4.1 ^n^), an NPZD-type ^o^ ocean ecosystem, and a terrestrial ecosystem component dealing with dynamic vegetation (SEIB-DGVM ^p^) [29].
Norwegian Climate Centre	Norway	NORESM1-M	Based on the CCSM4 ^r^, coupled by CAM4-Oslo ^s^ (Atmosphere), CLM4 ^s^ (Land), CICE4 ^t^ (Sea ice) and an isopycnic coordinate ocean general circulation model (Ocean) [30].

^a^ Climate Model, version 2.1. ^b^ Atmospheric Model, version 2. ^c^ Land Model, version 3.0. ^d^ GFDL Sea Ice Simulator. ^e^ Modular Ocean Model, version 4p1. ^f^ Hadley Centre Global Environmental Model-Atmosphere-Ocean. ^g^ Top-down Representation of Interactive Foliage and Flora Including Dynamics. ^h^ Diatom version of the Hadley Centre Ocean Carbon Cycle model. ^i^ UK Chemistry and Aerosols model. ^j^ Laboratoire de Météorologie Dynamique atmospheric general circulation model with zooming capability, version 5A. ^k^ Organizing Carbon and Hydrology in Dynamic Ecosystems. ^l^ Two-level thermodynamic-dynamic sea-ice model. ^m^ Nucleus of European Modelling of the Ocean, version 3.2. ^n^ Global chemical model of the troposphere, for study of atmospheric environment and radiative forcing, version 4.1. ^o^ Nutrient Phytoplankton Zooplankton Detritus. ^p^ Spatially Explicit Individual Based–Dynamic Global Vegetation Model. ^q^ Community Climate System Mode, version 4. ^r^ Community Atmosphere Model, Version Oslo. ^s^ Community Land Model, version 4. ^t^ Los Alamos National Laboratory sea ice model, version 4.

**Table 3:** Definition of extreme precipitation indices in this study.

Index	Definition	Units
AMX1p	Annual maximum 1-day precipitation	mm
AMX3p	Annual maximum 3-day precipitation	mm
AMX7p	Annual maximum 7-day precipitation	mm
AMX1d	Annual maximum 1-day discharge	m^3^/s
AMX3d	Annual maximum 3-day discharge	m^3^/s
AMX7d	Annual maximum 7-day discharge	m^3^/s

**Table 4:** Performance of the XAJ model for daily discharge simulation in Huatan and Pingshan.

Station	Period		*E_NS_*	*R* ^2^
Huatan	Calibration	1977–1990	0.852	0.857
	Validation	1991–2000	0.848	0.868
Pingshan	Calibration	1961–1990	0.841	0.854
	Validation	1991–2000	0.838	0.852

## References

[1] Winsemius H.C., Aerts J.C.J.H., Beek L.P.H.V., Bierkens M.F.P., Bouwman A., Jongman B. (2016). Global drivers of future river flood risk. Nat. Clim. Chang..

[2] Allen M.R., Ingram W.J. (2002). Constraints on future changes in climate and the hydrologic cycle. Nature.

[3] Dankers R., Feyen L. (2008). Climate change impact on flood hazard in Europe: An assessment based on high resolution climate simulation. J. Geophys. Res. Atmos..

[4] Pfahl S., O’Gorman P.A., Fischer E.M. (2017). Understanding the regional pattern of projected future changes in extreme precipitation. Nat. Clim. Chang..

[5] Apurv T., Mehrotra R., Sharma A., Goyal M.K., Dutta S. (2015). Impact of climate change on floods in the Brahmaputra basin using CMIP5 decadal predictions. J. Hydrol..

[6] Huang S., Krysanova V., Hattermann F. (2015). Projections of climate change impacts on floods and droughts in Germany using an ensemble of climate change scenarios. Reg. Environ. Chang..

[7] Duan J.G., Bai Y., Dominguez F., Rivera E., Meixner T. (2017). Framework for incorporating climate change on flood magnitude and frequency analysis in the Upper Santa Cruz River. J. Hydrol..

[8] Prudhomme C., Davies H. (2009). Assessing uncertainties in climate change impact analyses on the river flow regimes in the UK. Part 2: Future climate. Clim. Chang..

[9] Jung I.W., Chang H., Moradkhani H. (2011). Quantifying uncertainty in urban flooding analysis considering hydro-climatic projection and urban development effects. Hydrol. Earth Syst. Sci..

[10] Kingston D.G., Taylor R.G. (2010). Sources of uncertainty in climate change impacts on river discharge and groundwater in a headwater catchment of the Upper Nile Basin, Uganda. Hydrol. Earth Syst. Sci..

[11] Wilby R.L., Harris I. (2006). A framework for assessing uncertainties in climate change impacts: Low-flow scenarios for the River Thames, UK. Water Resour. Res..

[12] Eisner S., Voss F., Kynast E. (2012). Statistical bias correction of global climate projections-consequences for large scale modeling of flood flows. Adv. Geosci..

[13] Gaur A., Simonovic S.P. (2015). Towards Reducing Climate Change Impact Assessment Process Uncertainty. Environ. Process..

[14] Kiktev D., Sexton D.M.H., Alexander L., Folland C.K. (2003). Comparison of modeled and observed trends in indices of daily climate extremes. J. Clim..

[15] Schaeffer M., Selten F.M., Opsteegh J.D. (2005). Shifts of means are not a proxy for changes in extreme winter temperatures in climate projections. Clim. Dyn..

[16] Anandhi A., Nanjundiah R.S. (2015). Performance evaluation of AR4 Climate Models in simulating daily precipitation over the Indian region using skill scores. Theor. Appl. Climatol..

[17] Perkins S.E., Pitman A.J., Holbrook N.J., Mcaneney J. (2007). Evaluation of the AR4 climate models’ simulated daily maximum temperature, minimum temperature, and precipitation over Australia using probability density functions. J. Clim..

[18] Zhang Z., Klaus F., Jiang T., Zhang J. (2008). Projection of future precipitation extremes in the Yangtze River Basin for 2001–2050. Adv. Clim. Chang. Res..

[19] Yuan Z., Yang Z., Yan D., Yin J. (2017). Historical changes and future projection of extreme precipitation in China. Theor. Appl. Climatol..

[20] Yuan Z., Xu J., Chen J., Huo J., Yu Y., Locher P., Xu B. (2017). Drought Assessment and Projection under Climate Change: A Case Study in the Middle and Lower Jinsha River Basin. Adv. Meteorol..

[21] Liu H., Lan H., Liu Y., Zhou Y. (2011). Characteristics of spatial distribution of debris flow and the effect of their sediment yield in main downstream of Jinsha River, China. Environ. Earth Sci..

[22] Vuuren D.P.V., Edmonds J., Kainuma M., Riahi K., Thomson A., Hibbard K., Hurtt G.C., Kram K., Krey V., Lamarque J. (2011). The representative concentration pathways: An overview. Clim. Chang..

[23] Warszawski L., Frieler K., Huber V., Piontek F., Serdeczny O., Schewe J. (2014). The Inter-Sectoral Impact Model Intercomparison Project (ISI–MIP): Project framework. Proc. Natl. Acad. Sci. USA.

[24] Weedon G.P., Gomes S., Viterbo P., Shuttleworth W.J., Blyth E., Österle H., Adam J.C., Bellouin J.N., Boucher O., Best M. (2012). Creation of the WATCH forcing data and its use to assess global and regional reference crop evaporation over land during the twentieth century. J. Hydrometeorol..

[25] Hempel S., Frieler K., Warszawski L., Schewe J., Piontek F. (2013). A trend-preserving bias correction—The ISI-MIP approach. Earth Syst. Dyn..

[26] Dunne J.P., John J.G., Adcroft A.J., Griffies S.M., Hallberg R.W., Shevliakova E., Stouffer R.J., Cooke W., Dunne K.A., Harrison M.J. (2012). GFDL’s ESM2 Global Coupled Climate–Carbon Earth System Models. Part I: Physical Formulation and Baseline Simulation Characteristics. J. Clim..

[27] Jones C.D., Hughes J.K., Bellouin N., Hardiman S.C., Jones G.S., Knight J., Liddicoat S., O’Connor F.M., Andres R.J., Bell C. (2011). The HadGEM2-ES implementation of CMIP5 centennial simulations. Geosci. Model Dev..

[28] Persechino A., Mignot J., Swingedouw D., Labetoulle S., Guilyardi E. (2013). Decadal predictability of the Atlantic meridional overturning circulation and climate in the IPSL-CM5A-LR model. Clim. Dyn..

[29] Watanabe S., Hajima T., Sudo K., Nagashima T., Takemura T., Okajima H., Nozawa T., Kawase H., Abe M., Yokohata T. (2011). MIROC-ESM 2010: Model description and basic results of CMIP5-20c3m experiments. Geosci. Model Dev..

[30] Bentsen M., Bethke I., Debernard J.B., Iversen T., Kirkevåg A., Seland Ø., Drange H., Roelandt C., Seierstad I.A., Hoose C. (2013). The Norwegian Earth System Model, NorESM1-M—Part 1: Description and basic evaluation of the physical climate. Geosci. Model Dev..

[31] Zhao R.J., Liu X.R., Singh V.P. (1995). The Xinanjiang model. Comput. Models Watershed Hydrol..

[32] Gan T.Y., Dlamini E.M., Biftu G.F. (1997). Effects of model complexity and structure, data quality, and objective functions on hydrologic modeling. J. Hydrol..

[33] Todini E. (1996). The ARNO rainfall-runoff model. J. Hydrol..

[34] Yao C., Zhang K., Yu Z., Li Z., Li Q. (2014). Improving the flood prediction capability of the Xinanjiang model in ungauged nested catchments by coupling it with the geomorphologic instantaneous unit hydrograph. J. Hydrol..

[35] Zhang C., Wang R., Meng Q. (2015). Calibration of conceptual rainfall-runoff models using global optimization. Adv. Meteorol..

[36] Hargreave G.H., Samani Z.A. (1982). Estimating potential evapotranspiration. J. Irrig. Drain. Div..

[37] Nash J.E., Sutcliffe J.V. (1970). River flow forecasting through conceptual models part I-A discussion of principles. J. Hydrol..

[38] Tao Y., Shao Q.X., Hao Z.C., Xi C., Zhang Z.X., Xu C.Y., Sun L. (2010). Regional frequency analysis and spatio-temporal pattern characterization of rainfall extremes in the Pearl River Basin, China. J. Hydrol..

[39] Leclerc M., Ouarda T.B. (2017). Non-stationary regional flood frequency analysis at ungauged sites. J. Hydrol..

[40] Jeong J.H., Kannan N., Arnold J., Glick R., Gosselink L., Srinivasan R. (2010). Development and integration of sub-hourly rainfall–runoff modeling capability within a watershed model. Water Resour. Manag..

[41] Chau K.W., Wu C., Li Y. (2005). Comparison of several flood forecasting models in Yangtze River. J. Hydrol. Eng..

[42] Chen L., Guo S. (2019). Flood coincidence risk analysis using multivariate copula functions. J. Hydrol. Eng..

[43] Huang J., Zhang J., Zhang Z., Sun S., Yao J. (2012). Simulation of extreme precipitation indices in the Yangtze River basin by using statistical downscaling method (SDSM). Theor. Appl. Climatol..

[44] Su B., Huang J., Zeng X., Gao C., Jiang T. (2017). Impacts of climate change on streamflow in the Upper Yangtze River Basin. Clim. Chang..

[45] Gu H., Yu Z., Wang G., Wang J., Ju Q., Yang C., Fan C. (2015). Impact of climate change on hydrological extremes in the Yangtze River Basin, China. Stoch. Environ. Res. Risk Assess..

[46] Bell V.A., Kay A.L., Davies H.N., Jones R.G. (2016). An assessment of the possible impacts of climate change on snow and Peak River flows across Britain. Clim. Chang..

[47] Kay A.L., Davies H.N., Bell V.A., Jones R.G. (2009). Comparison of uncertainty sources for climate change impacts: Flood frequency in England. Clim. Chang..

[48] Teng J., Vaze J., Chiew F.H.S., Wang B., Perraud J.M. (2011). Estimating the relative uncertainties sourced from GCMs and hydrological models in modelling climate change impact on runoff. J. Hydrometeorol..

[49] Xu Y.P., Zhang X., Ran Q., Tian Y. (2013). Impact of climate change on hydrology of upper reaches of Qiantang River Basin, East China. J. Hydrol..

[50] Merz R., Parajka J., Blöschl G. (2011). Time stability of catchment model parameters: Implications for climate impact analyses. Water Resour. Res..

[51] Costabile P., Costanzo C., Macchione F. (2013). A storm event watershed model for surface runoff based on 2D fully dynamic wave equations. Hydrol. Process..

[52] Bellos V., Tsakiris G. (2016). A hybrid method for flood simulation in small catchments combining hydrodynamic and hydrological techniques. J. Hydrol..

[53] Xia X., Liang Q., Ming X., Hou J. (2017). An efficient and stable hydrodynamic model with novel source term discretization schemes for overland flow and flood simulations. Water Resour. Res..

